# Treatment of periorbital hyperpigmentation using sublative fractional radiofrequency (SFR)

**DOI:** 10.1111/srt.13467

**Published:** 2023-09-13

**Authors:** Mohammad Ali Nilforoushzadeh, Maryam Heidari‐Kharaji, Tannaz Fakhim, Elham Torkamaniha, Sepideh Tehrani, Shohreh Delavar, Shohreh Rafiee, Maryam Nouri, Niloufar Najar Nobari, Mohammadhasan Shahverdi

**Affiliations:** ^1^ Skin and Stem Cell Research Center Tehran University of Medical Sciences Tehran Iran; ^2^ Skin Repair Research Center Jordan Dermatology and Hair Transplantation Center Tehran Iran; ^3^ Institut National de la Recherche Scientifique (INRS)‐Centre Armand‐Frappier Santé Biotechnologie (CAFSB) Laval Quebec Canada; ^4^ Department of Microbial Biotechnology Islamic Azad University Kish Branch Iran; ^5^ Department of Dermatology Amir Al Momenin Teaching Hospital Tehran Iran; ^6^ Tehran Medical Branch School of Medicine Islamic Azad University Tehran Iran; ^7^ Islamic Azad University of Medical Sciences Shariati St Tehran Iran; ^8^ Department of Dermatology, Rasool Akram Medical Complex Iran University of Medical Sciences Tehran Iran

**Keywords:** dark eye circle, nonablative radiofrequency (sfr), periorbital hyperpigmentation (poh), treatment

## Abstract

**Background:**

Periorbital hyperpigmentation (POH) is a common cosmetic concern. Numerous techniques of treatment have been assessed with variable results.

**Aim:**

The purpose of this research is to assess the efficacy of non‐ablative radiofrequency, Sublative fractional Radiofrequency (SFR) on POH treatment.

**Methods:**

In this research study, nine patients with POH and the age range of 25−57 years, were enrolled. The patients were treated by non‐ablative radiofrequency SRF. The outcomes were assessed by biometric assessment. The skin lightness and melanin content of the periorbital skin were assessed by colorimeter and Mexameter. Skin elasticity was assessed by Cutometer. The skin ultrasound imaging system was used to evaluate the diameter and density of the epidermis and dermis. Visioface was used to evaluate the skin color and wrinkles. Also, patient's satisfaction and physician's assessment were assessed.

**Results:**

The results showed that the lightness and elasticity of the periorbital skin were significantly increased after treatment. Also, the melanin content of the skin was decreased. The denser skin layers were seen in both dermis and epidermis. The Visioface results displayed the reduction in the percent change of the skin color and wrinkle (*p* < 0.05). Similarly, the physician and patients’ assessment confirmed the outcomes. No serious adverse effect was reported.

**Conclusion:**

In conclusion, the SFR technique is an effective and satisfactory therapeutic choice for treatment of POH.

## 1 INTRODUCTION

Dark circles under the eyes is called periorbital hyperpigmentation (POH), which is a common concern in adult and young patients in both sexes with any type of skin. POH might look as symmetrical hyperpigmented spots around the eye and can affect one area of the eye more than the other one. It can also be present on the lower and upper eyelid and can extend to the upper of the nose.^1^ The POH etiology is multifactorial. Some factors that contribute to POH are as follows: skin laxity, tear trough and volume loss, prominent vasculature, allergy, and atopic dermatitis.^1^ In some studies, contact‐free hyperspectral imaging showed that melanin is the main chromophore in POH and hemoglobin oxygen saturation is secondary.^2^ The results of the Kikuchi et al. (2013) study that used a contact spectrophotometer confirmed that melanin content increased, in the POH patients.^3^ Beauty can affect a person's quality of life by increasing the self‐confidence. On the other hand, the degree of fatigue and age is related to the condition of the skin around the eyes. The dark circle around the eyes has the key role in creating a sad, tired, old appearance, and force high cosmetic costs on the person.^4^, ^5^ POH can cause the tired appearance and various equipment and tools have been developed to improve the disorder like fillers, chemical treatment, fat injection, and more invasive interventions such as various surgical procedure.^4^ The laser therapy is a new method for the POH treatment.^6^, ^7^ Lasers have selective ability to target endogenous chromophores.^8^ Laser therapy is commonly perfect for the treatment of larger and deep facial vessels because of the superior penetration of laser energy.^1^ In non‐ablative fractional laser therapy, fragmental thermal damage is stimulated in exposed skin. Radiofrequency (RF) is rapidly EVOLVING field update.^9^, ^10^, ^11^ In treatment by RF, the usage of radiofrequency electromagnetic waves makes local hyperthermia via changing electrical energy to intracellular heat with individual skin structures absorption. The dose of light and wavelength are regulated to change the skin‐coloring, decrease erythema, and to the photo‐rejuvenation of ageing. This treatment stimulates a thermal response in the dermis, therefore promoting the remodeling of connective tissue and the new collagen formation, thereby improving the skin elasticity.^12^ The purpose of this study is to assess the effect of sublative fractional radiofrequency as non‐ablative radiofrequency (SFR) in the treatment of POH (melanin, pigmentation, elasticity, and erythema in the eyes area have been evaluated). Furthermore, we also assessed the decrease in the number of wrinkles and changes related to other skin defects.

## 2 METHODS

### 2.1 Participants

In the current study, nine patients with POH (average age: 36) were involved. Patients were treated with SFR RF laser. After description of the route of treatment, consent was gained from each subject for joining and photography.

### 2.2 Inclusion and exclusion criteria

Inclusion criteria were age more than 18 years, without any infections in the region, no untreated comorbidities, and informed patient agreement.

The exclusion criteria were, local skin disease, pregnancy or lactation, collagen vascular diseases, local or systemic infections, and the persons who had laser therapy in the previous 1 year in that region.

### 2.3 Study treatment

All patients receive non‐ablative SFR RF (LUTRONIC, INFINI, Level 3). Suitable time and heating temperature according to the pain tolerance of each patient was used during the procedure [level 4–5, time (ms): 60 for each eye, shot count: 10–12 for each eye]. The patients receive the treatment every 3 weeks three to five times according to the patients age, sex, and the amount of darkness. Conductor gel was used throughout the process.

### 2.4 Clinical evaluation of outcomes

### 2.5 Biometric assessment

For all patients, biometric characteristics were assessed before and 6 months after the last treatment session, by, Cutometer, and Visioface, Mexameter, Colorimeter (Germany), and the skin ultrasound imaging system (TPM, Germany).

### 2.6 Colorimeter

The colorimeter was used to assess the skin lightness of the periorbital area. The colorimeter automatically calculated differences in pores and color and gave numeric results. The lighter skin has a lower number.

### 2.7 Cutometer

The Cutometer is a noninvasive tool that utilized to assess skin biomechanical possessions. It appraises skin elasticity by negative pressure that distorts the skin mechanically. In this study, the three parameters R2, R5, and R7 were analyzed. For the evaluation of skin aging and elasticity, R2 and R7 are applied as key Cutometer parameters. R7 is related to biological elasticity. R7 is characterized with the immediate retraction ratio to the final distension, (Ur/Uf). Aging causes the reduction in R7 parameter. Furthermore, we evaluated factors R2 and R5. R2 is related to skin visco‐elasticity, containing viscous deformation and is characterized with the ratio of the ability of the skin re‐bend to the final distension (Ua/Uf). R5 refers to net elasticity and is represented by the ratio of elastic portion of the suction stage to fast recovery throughout relaxation stage (Ur/Ue).

### 2.8 Mexameter and Visioface

Mexameter MX 18 is a noninvasive tool utilized to evaluate the skin melanin concentration and hemoglobin. The data is prepared by the light absorption phenomenon. Three different wavelengths emit with a special probe and the reflected skin light computes by the receptor. Since total of the produced light is exactly measured, it is probable to compute the total of light that absorbed with the skin. Wavelengths were selected to attain diverse levels of absorption with melanin and hemoglobin. The intensity of the erythema in the treated area was evaluated by the Mexameter (MX 18 probe) as well. Lower amounts revealed the lighter skin. Also, the changes in skin wrinkle and color were evaluated by Visoface.

### 2.9 Skin ultrasound imaging system

The skin ultrasound imaging system (HFUS: high‐frequency ultrasound; TPM, Germany; DUB Skin Scanner; 75 MHZ probe) was estimated the epidermis and dermis density and diameter. Higher density shows a higher collagen amount of dermis and showing well patient situations.

### 2.10 Digital photographs, patient's satisfaction, and physician assessment

Digital photographs were taken before and after treatment. The patient's satisfaction was evaluated as follows: No satisfaction, slightly satisfied, moderately satisfied, and well satisfied.

Dermatologists’ assessment was evaluated regarding to VAS score.

### 2.11 Statistical analysis

Results are shown as a mean and standard deviation (mean ± SD). For statistical analysis, SPSS 15.0 statistical software (SPSS) was utilized. Also, significance of differences between before and after treatment was calculated with the one‐way ANOVA. A *p* value **<** 0.05 was considered as significant.

## 3 RESULTS

### 3.1 Biometric characteristic results

**FIGURE 1:**
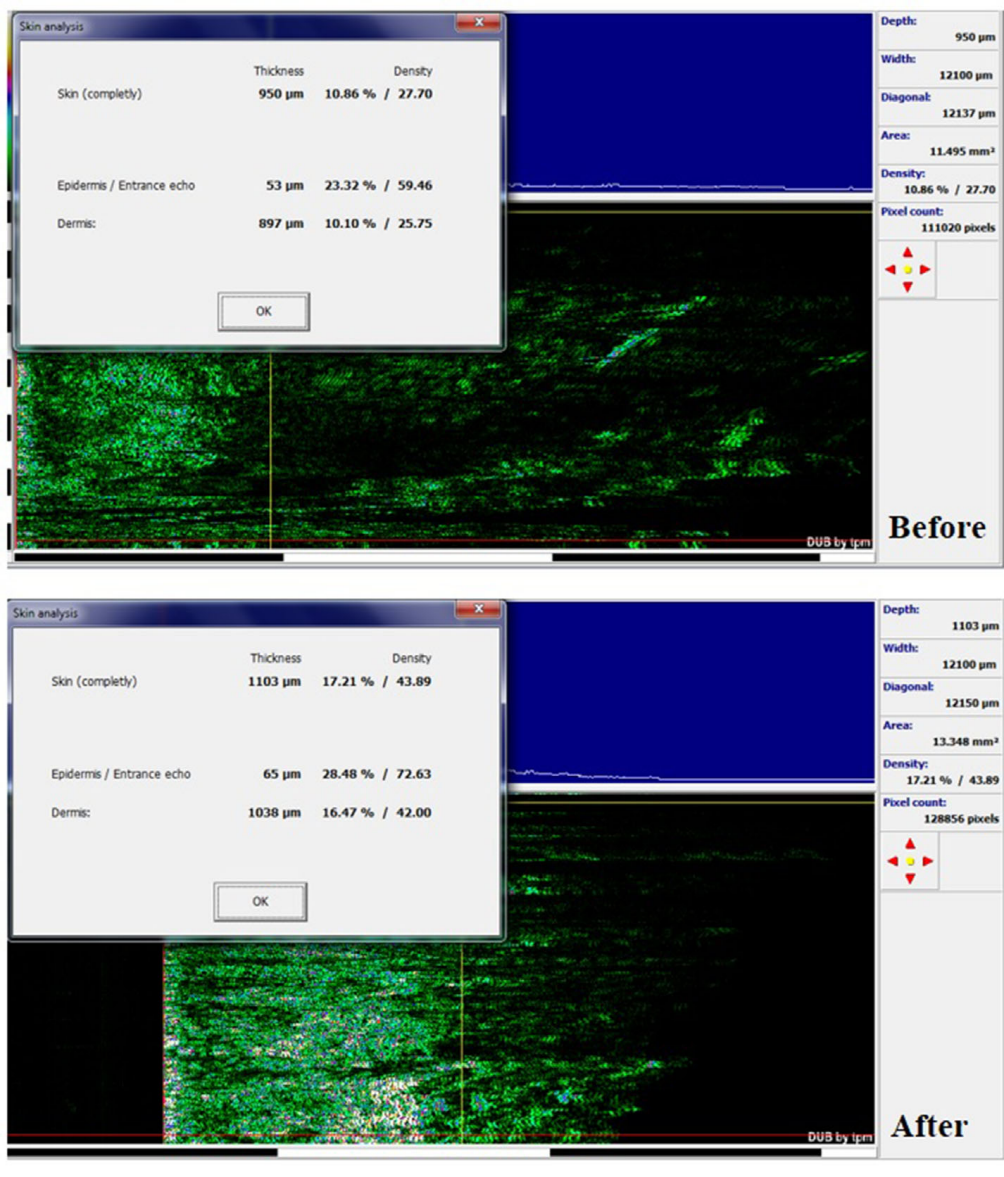
Skin analysis with ultrasonography. The skin density and thickness, before and 6 months after the last SFR treatment session.

**FIGURE 2:**
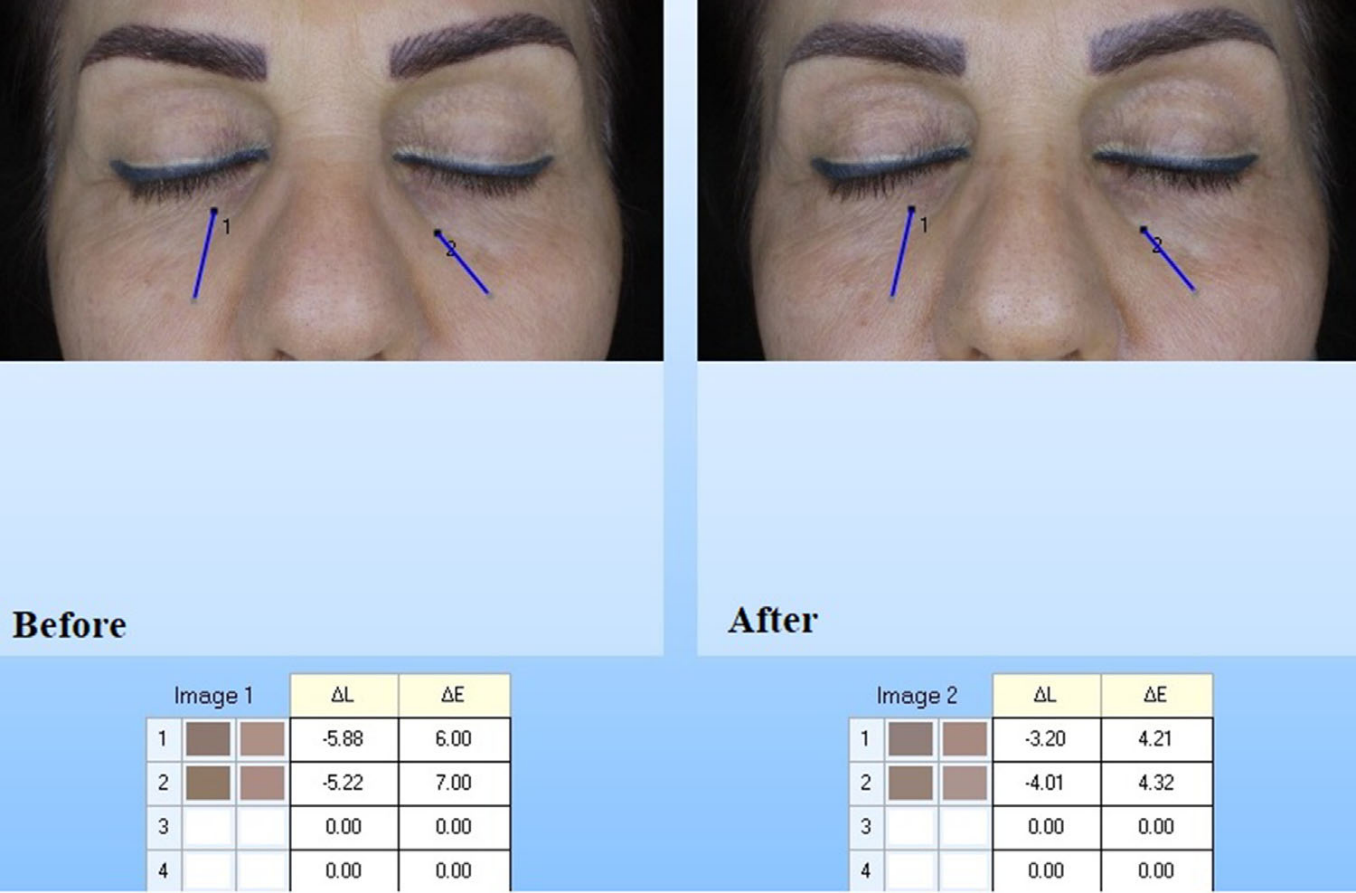
Periorbital visioface color analysis before and 6 months after the last SFR treatment session.

**FIGURE 3:**
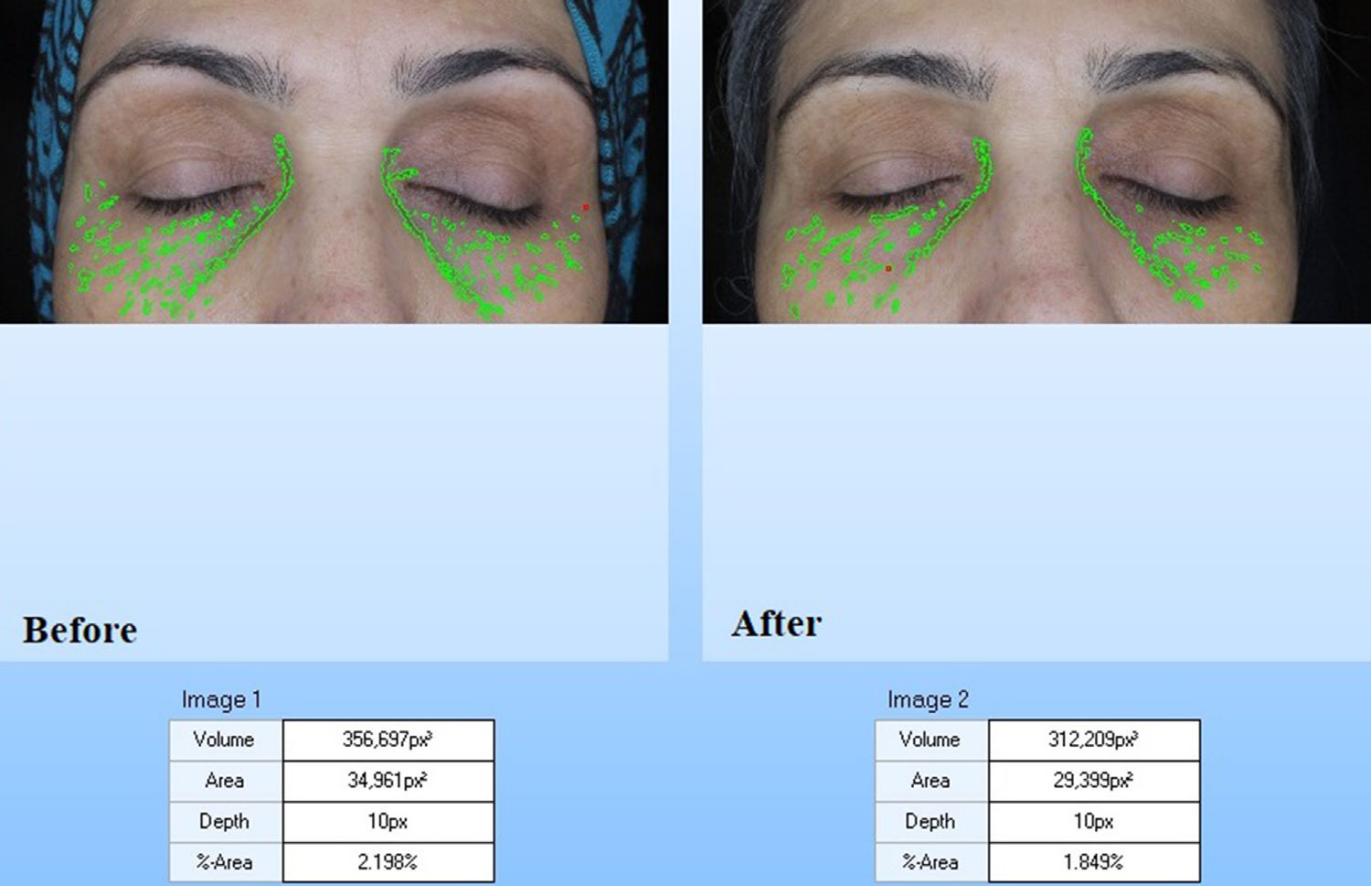
Periorbital visioface wrinkle analysis before and 6 months after the last SFR treatment session.

The biometric evaluation results are presented in Table 1. According to the mexameter results, the melanin content of the periorbital skin was significantly decreased before and after treatment (246.21 ± 30.04) and (150.05 ± 21.24), respectively (Table 1) (*p* < 0.05), and the percentage of change was evaluated as (40.41 ± 7.14) (*p* < 0.05) (Table 1). Furthermore, the colorimeter results indicated that, the lightness of the periorbital skin in the patients before and after treatment was 19.25 ± 5.35 and 27.35 ± 6.23, respectively (Table 1). The lightness of the periorbital skin was significantly increased after treatment (*p* < 0.05). The percentage of change was 42.18 ± 6.17 (Table 1) (*p* < 0.05). Moreover, the percentage of change in erythema was not significant before and after treatment (1.7 ± 0.51) (*p* < 0.05) (Table 1). So, no significant side effect was seen after treatment. According to the Cutometer outcomes, a significant increase was seen in the skin elasticity (*p* < 0.05). Also, the periorbital skin ultrasound showed denser skin layers after treatment in the dermis and epidermis (Table 1), and the percentages of changes were significant in skin density and thickness (*p* < 0.05) (Figure 1). The results of visioface showed that the color of the treated area was 11.13 ± 3.23 and 7.15 ± 1.20 before and after treatment (Table 1 and Figure 2). There was a significant change in the color of the periorbital after treatment (Figure 2), *p* < 0.05. Similarly, the visioface results displayed the significant decrease in the skin wrinkle 6 months after last treatment (Table 1, Figure 3). The percentage of change in the wrinkle area and volume were 25.44 ± 7.03 and 22.16 ± 4.12, respectively (Table 1).

**TABLE 1:** Comparing biometric characteristics of the periorbital dark circles in patients, before and 6 months after the last treatment session.

	Measured values			
	Before	After	Percent change	*p value*
Mexameter				
Melanin	246.21 ± 30.04	150.05 ± 21.24	40.41 ± 7.14	< 0.05
erythema	337.11 ± 55.06	343.54 ± 43.51	1.7 ± 0.51	0.603
Skin lightness^a^	19.25 ± 5.35	27.35 ± 6.23	42.18 ± 6.17	< 0.05
Skin ultrasonography				
Skin density (μm)	13.98 ± 4.11	18.99 ± 6.16	38.01 ± 9.16	< 0.05
Skin thickness (μm)	838.19 ± 40.02	1216.36 ± 100.07	45.02 ± 11.01	< 0.05
Epidermis density	26.22 ± 8.76	33.19 ± 6.15	27.22 ± 8.11	< 0.05
Epidermis thickness	53.10 ± 11.21	65.63 ± 10.15	22.26 ± 5.32	< 0.05
Dermis density	12.92 ± 2.18	17.83 ± 5.94	41.45 ± 10.16	< 0.05
Dermis thickness	898.15 ± 195.17	1137.50 ± 176.01	26.61 ± 8.51	< 0.05
Elastisity^b^				
R2	0.29 ± 0.05	0.41 ± 0.07	41.09 ± 7.12	< 0.05
R5	0.342 ± 0.02	0.446 ± 0.01	29.73 ± 7.08	< 0.05
R7	0.194 ± 0.01	0.276 ± 0.05	42.53 ± 13.16	< 0.05
Visioface				
color (ΔE^c^ ^)^	11.13 ± 3.23	7.15 ± 1.20	36.14 ± 8.30	< 0.05
Wrinkle				
Area	34.9 ± 6.07	26.3 ± 5.01	25.44 ± 7.03	< 0.05
volume	295 ± 6.12	230.7 ± 2.13	22.16 ± 4.12	< 0.05

The data was shown as mean ± SD. The *p* < 0.05 was considered as statistically significant in all the tests.

^a^ Skin lightness measured by Colorimeter (CYL, RGB).

^b^ Elasticity of the skin measured by Cutometer (mm/time).

^c^ The color difference.

### 3.2 Patients satisfaction and physicians’ assessment results

**FIGURE 4:**
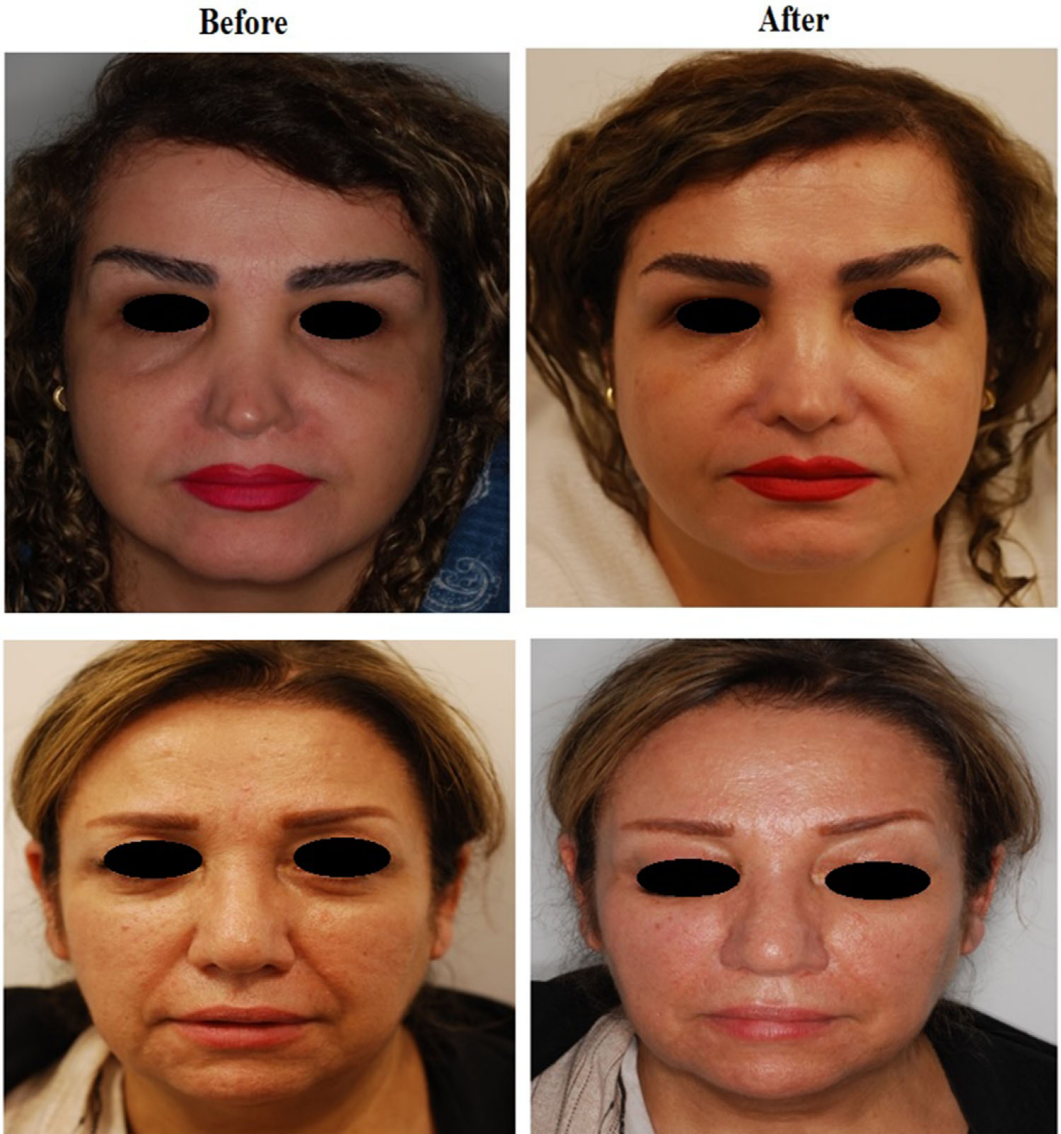
The study patients before and after 6 months of last treatment session.

The patients’ satisfaction results were as follows: 20% slightly satisfied, 30% moderately satisfied, and 50% well satisfied (Table 2). the physicians’ assessments are shown in Table 3 (*p* < 0.05). The physicians’ assessments results showed a significant change before and after treatment (*p* < 0.05). The results of digital photographs are shown in Figure 4.

**TABLE 2:** Patient satisfaction 6 months after the last treatment session.

Valid	Frequency	Percentage
No satisfaction	0	0
Slightly satisfied	2	20
Moderately satisfied	3	30
Well satisfied	5	50
Total	10	100

**TABLE 3:** Physician assessment regarding to VAS 6 months after the last treatment session.

	Physicians’ assessment	
VAS Score	Mean ± SD (Range)	*p value*
Before treatment	8.0 ± 1.1 (6–9)	<0.05
After treatment	4.5 ± 1.0 (1–7)	

The data was shown as mean ± SD.

## 4 DISCUSSION

The increase of dark circles under the eyes at any age is a major aesthetic concern as it may make a person appear sad, old, tired, and stressed.^4^ One of the first part of the face show the signs of elderly is the periorbital area. Even slight changes in its structure or volume can change the patient's emotions and health, possibly reducing their well‐being and confidence.^13^ Because of the complexity of POH pathogenesis, several methods are evaluated for treatment. Recently, laser therapy for skin disorder treatment is developed.^14^, ^15^, ^16^, ^17^, ^18^, ^19^, ^20^ In the study of Nilforoushzadeh et al., the effectiveness of the combination therapy with fractional Er‐YAG laser and platelet‐rich plasma (PRP) was assessed in treatment of periorbital dark circles and they reported a significant result after treatment.^7^ In another study, the effect of carboxy therapy and fractional Q‐switched ND:YAG laser were evaluated on periorbital dark circles treatment.^6^ The purpose of current study was to assessment the pigmentation treatment results after using, non‐ablative RF (SFR). The outcomes of mexametric records were statistically significant after 6 months. Other authors used Mexameter to measure the decrease of skin hyperpigmentation.^6^, ^7^, ^21^, ^22^, ^23^ In our study, measurements were done at the same locations for both measurements of the Mexameter and Cutometer probe. In study of Li et al., women with melasma were treated with IPL and Mexameter results revealed a significant decrease of pigmentation in the melasma lesions^24^. Shin et al. utilized Mexameter as a noninvasive and objective tool for assessment of photorejuvenation effect of IPL treatment in the skin of 26 women.^25^ Our colorimeter results showed that the lightness of the treated area was significantly increased after treatment (*p* < 0.05). The Cutometer outcomes displayed a significant increase in the elasticity of the periorbital skin. Moreover, the erythema was not significantly changed after treatment. In Choi et al. study, the skin of 97 females was analyzed by the Cutometer, Visioscan, and Corneometer probes. According to their results, elastic recovery ratio (R7) reduced with age.^26^ Other studies confirm the results of R7 analysis gained by Choi .^27^, ^28^, ^29^ Our investigation outcomes are similarly confirmed by the above‐declared reports. Our outcomes regarding R7 factor showed that non‐ablative RF treatment are related to the increase of the skin elasticity. Our results showed that the SRF as a non‐ablative technique is an effective method for the reduction in periorbital pigmentation with no significant side effect (like erythema) after treatment.

## 5 CONCLUSION

Periorbital hyperpigmentation is a common cosmetic concern. Numerous techniques of treatment have been assessed with variable results. Our results showed that the SRF as non‐ablative technique is an effective method for the reduction in periorbital pigmentation with no significant side effect (such as erythema) after treatment.

### 5.1 Study limitation

The use of a small sample size in the current study can be considered as a study limitation.

## CONFLICT OF INTEREST STATEMENT

The authors declare that they have no conflict of interest.

## ETHICS STATEMENT

Informed consent was obtained from all the patients. All patients were provided with a complete description of the study design, purpose, and probable outcomes. All the patients were checked before and 6 months after the last session of treatment.

## Data Availability

The data that support the findings of this study are available from the corresponding author upon reasonable request.
